# Supplementary material for: Genomic Mapping of Epidermal Growth Factor Receptor and Mesenchymal–Epithelial Transition-Up-Regulated Tumors Identifies Novel Therapeutic Opportunities
Source: Cancers (Basel). 2023 Jun 20;15(12):3250. doi: 10.3390/cancers15123250 (PMC10297015; doi:10.3390/cancers15123250)
Supplement: Supplementary file 1 [file cancers-15-03250-s001.zip › cancers-2441514-supplementary.pdf]

Tumor	Gen	Treatment	FDR (%)	HR	p value
Bladder (n = 90)	EGFR	anti-PD-1	100	0.52	0.1866
		anti-PD-L1	100	0.62	0.3575
	MET	anti-PD-1	100	0.47	0.0926
		anti-PD-L1	100	0.52	0.1493
Esophageal adenocarcinoma (n = 103)	EGFR	anti-PD-1	-		
		anti-PD-L1	1	0.27	0.0003
	MET	anti-PD-1	-		
		anti-PD-L1	100	0.43	0.0758
Glioblastoma (n = 28)	EGFR	anti-PD-1	20	2.96	0.0193
		anti-PD-L1	-		
	MET	anti-PD-1	100	2.26	0.0816
		anti-PD-L1	-		
Hepatocellular carcinoma (n = 22)	EGFR	anti-PD-1	-		
		anti-PD-L1	-		
	MET	anti-PD-1	-		
		anti-PD-L1	-		
HNSCC (n = 110)	EGFR	anti-PD-1	-		
		anti-PD-L1	-		
	MET	anti-PD-1	-		
		anti-PD-L1	-		
Melanoma (n = 570)	EGFR	anti-PD-1	over 50	0.67	0.0109
		anti-PD-L1	-		
	MET	anti-PD-1	20	1.7	0.0014
		anti-PD-L1	-		
NSCLC (n = 21)	EGFR	anti-PD-1	100	1.64	0.3519
		anti-PD-L1	-		
	MET	anti-PD-1	-		
		anti-PD-L1	-		
NSLC (n = 22)	EGFR	anti-PD-1	-		
		anti-PD-L1	-		
	MET	anti-PD-1	-		
		anti-PD-L1	-		
Urothelial (n = 348)	EGFR	anti-PD-1	-		
		anti-PD-L1	100	1.26	0.0743
	MET	anti-PD-1	-		
		anti-PD-L1	over 50	1.47	0.0168

Supplementary table 1 Kaplan-Meier survival values of EGFR and MET in patients treated with anti PD1 and anti PD-L1. Table includes the fold discovery rate (FDR), the hazard ratio (HR), and p-value for several tumors. Studies with not enough samples to perform the analysis are marked with a hyphen.
